# Latency, tropism and genetic variation of Simian Foamy Virus in blood and saliva from infected Humans

**DOI:** 10.1186/1742-4690-11-S1-O71

**Published:** 2014-01-07

**Authors:** Réjane Rua, Edouard Betsem, Thomas Montange, Florence Buseyne, Antoine Gessain

**Affiliations:** 1 Unit of Epidemiology and Physiopathology of Oncogenic Viruses, Department of Virology, Institut Pasteur, France; 2 Centre National de la Recherche Scientifique (CNRS), UMR 3569, Paris, France; 3 Université Paris Diderot, Cellule Pasteur, Paris, France; 4 Faculty of Medicine and Biomedical Sciences, University of Yaounde I, Yaounde, Cameroon

## 

Simian foamy viruses (SFV) are widespread retroviruses among non-human primates (NHP). SFV actively replicate in the oral cavity of NHP and can be transmitted to humans through NHP bites, in whom they establish a persistent infection. We aimed to study three major properties of these zoonotic retroviruses: replicative status, tropism and variability. In 14 hunters from Cameroon previously shown to be infected with a gorilla SFV strain, viral DNA could be detected by quantitative polymerase chain reaction in most samples of peripheral blood mononuclear cells (PBMCs) and saliva. The SFV DNA levels were 7.1±6.0 SFV DNA copies/10^5^ cells in PBMCs and 2.4±4.3 SFV DNA copies/10^5^ cells in saliva. In contrast, no SFV RNA was detected by qRT-PCR in either PBMCs or saliva. PBMCs populations (T4, T8, B, NK lymphocytes and monocytes) were sorted with magnetic beads before quantification of SFV DNA. Our preliminary results showed the presence of SFV DNA in all PBMCs populations at different levels. We finally assessed the viral diversity *in vivo*. Although intra-individual SFV genetic variation was low (<0,5%) we detected some viral diversity in 3 out of 9 individuals. In one subject, genetic variation might be associated with coinfection with 2 SFV strains, while in the two other subjects, variations seemed to derive from APOBEC3 editing with a high rate of G-to-A substitutions. Our study demonstrates that SFV infection is mostly latent in PBMCs and in saliva. Such a scenario may explain the putative lack of secondary human-to-human transmissions of SFV.

